# Alterations in expression profile of iron-related genes in colorectal cancer

**DOI:** 10.1007/s11033-013-2659-3

**Published:** 2013-09-28

**Authors:** Katarzyna Hamara, Anna Bielecka-Kowalska, Karolina Przybylowska-Sygut, Andrzej Sygut, Adam Dziki, Janusz Szemraj

**Affiliations:** 1 Department of Medical Biochemistry, Medical University of Lodz, Mazowiecka 6/8, 92-215 Lodz, Poland; 2 Department of Clinical Chemistry and Biochemistry, Medical University of Lodz, Plac Hallera 1, 90-647 Lodz, Poland; 3 Department of General and Colorectal Surgery, Medical University of Lodz, Plac Hallera 1, 90-647 Lodz, Poland

**Keywords:** Colorectal adenocarcinoma, Iron metabolism, MiRNA expressing profile, Diagnosis of cancer

## Abstract

Iron can play a role in colorectal cancer (CRC) development. The expression of genes involved in iron metabolism and its regulation in CRC has not been investigated well. Also the correlation between the level of iron-related genes expression and cancer progression is not known. In this study we collected paired samples of primary adenocarcinoma and adjacent normal mucosa from 73 patients. We assessed the mRNA or miRNA levels of 21 genes and verify their association with clinicopathological characteristics of CRC patients. Our experiments revealed, that the level of divalent metal transporter 1 transcript is well correlated with mRNA levels of iron regulatory proteins (IRPs) in tumor specimens. We have shown, that IRP2 can also be engaged in the mRNA stabilization of other iron transporter–transferrin receptor 1 (TfR1) in early stage of disease, however, in more advanced stages of CRC, mRNA level of *TfR1* is related to miR-31 level. For the first time we have shown, that ferroportin concentration is significantly associated with miR-194 level, causing the reduction of this transporter amount in tumor tissues of patients with more advanced stages of CRC. We have also shown the alterations in expressing profile of miR-31, miR-133a, miR-141, miR-145, miR-149, miR-182 and miR-194, which were observed even in the early stage of disease, and identified a set of genes, which take place in correct assigning of patients in dependence of CRC stage. These iron-related genes could become potential diagnostic or prognostic indicators for patients with CRC.

## Introduction

Colorectal cancer (CRC) is the third most commonly diagnosed cancer in males and the second in females worldwide, and ranks fourth and third for cancer-related mortality among males and females, respectively [1]. There is a body of evidence for a role of iron in CRC development. Epidemiological studies revealed the association of elevated total body iron and a high dietary iron intake with CRC risk [2, 3]. It was also confirmed with the observation, that patients with *HFE* mutations, suffering from iron overload condition (hereditary hemochromatosis), had an increased risk of CRC [4]. Iron is widely involved in many important metabolic processes, such as electron transport, oxygen delivery, enzymes and/or coenzymes activity, and also DNA synthesis, which is intensified in proliferating cancer cells. On the other hand, many symptoms are associated with CRC, and iron deficiency anaemia (IDA) is a classical indicator of this malignancy. It is present in 11–57 % of cancers and is more common in patients with right-sided tumors (65–80 % have IDA) [5–7]. Anaemia is presumed to be a result of the chronic occult blood loss related to the presence of tumor in the right colon compared to the rectal bleeding of left sided tumors, which is detected sooner [8]. Thus, various perturbations of iron metabolism can be observed in patients with colorectal adenocarcinoma.

Iron is absorbed from the diet via the duodenal enterocytes. Free ferric iron is reduced to ferrous state by cytochrome b-like ferrireductase (Dcytb), and then transferred through the apical enterocyte membrane via divalent metal transporter 1 (DMT1). Iron can be bound to intestinal ferritin (Fn) and stored, or moved to the basolateral surface of the cell and exported by ferroportin (FPN1). After reoxidation to its ferric state by ferrooxidase hephaestin (Heph), iron is bound to serum transferrin (Tf) for distribution to tissues. On the surface of target cells, the diferric Tf is recognized by two highly specific transferrin receptors (TfRs), TfR1 and TfR2, which allow the cellular uptake of transferrin-bound iron by the receptor-mediated endocytosis. Acidification (pH 5.5) of the endosomes results in protein conformation changes following iron dissociation from Tf. Ferric iron is then reduced and transported from the endosome to the cytoplasm by DMT1. The Tf cycle is completed when the endosome fuses with the plasma membrane, returning apo-Tf to the circulation and TfR1 to the plasma membrane [9]. The target for transferrin-bound iron can be the bone marrow, where erythrocyte formation, heme synthesis and also iron utilization is performed, or hepatocytes, where iron is bound to Fn and stored. Mononuclear macrophages involved in the recycling of iron from senescent erythrocytes also accumulate iron. In case of iron depletion, iron release from the storage cells is mediated by FPN1 [10] and regulated via hepcidin [11].

Hepcidin, a key regulator of iron metabolism, can prevent cellular iron export by internalization and the lysosomal degradation of ferroportin. Thus, increased hepatic hepcidin synthesis in response to iron overload results in subsequent inhibition of iron release from duodenal enterocytes by limiting the ferroportin available on the cell surface. Conversely, decreased hepcidin production under iron limiting conditions enhances FPN1 expression and increases intestinal iron absorption [12]. Expression of hepcidin is homeostatically regulated by anaemia, hypoxia, inflammation [13], and also by the function of the hemochromatosis protein (HFE), hemojuvelin (HJV) and TfR2, as indicated by their mutation that decreased hepcidin expression [14–16]. The fact that HFE forms a protein complex with TfR [17] has led to the attractive hypothesis, that a soluble factor such as diferric Tf (which competes with HFE for TfR binding) might modulate HFE activity and regulate a potential pathway signaling to the hepcidin (*HAMP*) promoter [18]. To date, direct evidence supporting this hypothesis is lacking. Other data suggest that cell-associated HJV is required for normal expression of hepcidin in hepatocytes, and that soluble HJV negatively regulates hepcidin expression in hepatocytes [19].

Other regulatory system of iron metabolism is dependent on the activity of iron regulatory proteins (IRPs), IRP1 and IRP2, which control the posttranscriptional expression of genes modulating cellular iron uptake, export and storage. Under low iron conditions, both proteins bind to conserved, hairpin-like iron regulatory elements (IREs) present in the untranslated regions (UTRs) of target mRNAs. The IRE/IRP interactions result in stabilization and protection of mRNA from endonucleolytic degradation (when IRPs are bound to 3′-UTR of *TfR1* mRNA) or in specific inhibition of mRNA translation into protein (when bound to 5′-UTR of the H- and L-*Fn* mRNAs). Thus, iron-depleted cells increase their capacity of transferrin-bound iron uptake by TfR1 and minimize its sequestration into Fn stores. In contrast, in body iron excess condition IRPs are inactivated promoting *TfR1* mRNA degradation and *Fn* mRNA translation, which results in inhibition of further iron uptake and increased iron acquisition [20]. The presence of IREs in other iron-related mRNAs (*DMT1* and *FPN1*) suggests that their expression may be controlled by cellular iron content, but it has not been well understood yet [21, 22].

In the last few years new epigenetic regulators of iron metabolism have emerged. MicroRNAs, short noncoding RNAs, can control protein expression at the post-transcriptional level. The principal mechanism of miRNA regulation of gene expression is through the formation of a miRNA-induced silencing complex in the cytoplasm, which binds to 3′-UTRs of target transcripts, thereby leading to repression of protein translation or destabilization of the mRNAs with a significant implication in pathology, especially in cancers [23]. Deregulation of miRNA expression levels and some genetic alterations were demonstrated in various cancers, including colorectal adenocarcinoma [24, 25]. Investigations in tissue samples have identified altered expressions of miRNAs associated with tumorigenesis and tumor progression. Overexpression of some tumor-inducing or tumor-promoting miRNAs was demonstrated and these miRNAs became the candidate biomarkers for diagnostics or monitoring, offering relevant insights into tumorigenic mechanisms.

Proteins involved in iron metabolism are typically synthesized by hepatocytes. However, iron metabolism alters greatly in tumor cells and the up-regulation of TfRs on the cell surface and Fn are the most well known modifications [26]. The functions of iron-related proteins in association with colorectal adenocarcinoma have been poorly investigated. It was reported that CRC is related to overexpression of genes involved in iron import (*Dcytb*, *DMT1*, and *TfR1*) and decreased expression of genes involved in iron export (*FPN1* and *Heph*), which can promote iron storage in this tumor [27]. Recent studies have indicated, that hepcidin mRNA is overexpressed in 34 % of tumor tissues and is associated with iron deficiencies [28]. Hence, at the colonocyte level increased hepcidin levels may locally support iron accumulation promoting cancer progression. Hitherto, the alterations in expression of remaining genes involved in iron metabolism and its regulation in cancer tissues has not been investigated yet. Also the correlation between the level of iron-related genes expression and cancer progression in patients is not known.

The aim of this study was the verification of hypothesis: (1) whether the expression of analyzed genes is significantly different in tumor versus normal colon tissues from the same resection specimen in colorectal adenocarcinoma patients, (2) whether the alterations in genes expression appear in early stage of disease, (3) whether there is a correlation between expression of analyzed genes and cancer progression, (4) whether there is a correlation between the level of mRNA and homologous miRNA, and (5) whether there is a correlation between expression levels and single nucleotide polymorphisms of *HAMP* gene encoding hepcidin. To reach these goals we collected paired samples of primary cancer and adjacent normal mucosa from 73 CRC patients undergoing surgery. The mRNA or miRNA level of 21 genes (*DMT1*, *Fn*, *FPN1*, *HAMP*, *Heph*, *HFE*, *HJV*, *IRP1*, *IRP2*, *Tf*, *TfR1*, *TfR2*, miR-19a, miR-31, miR-133a, miR-141, miR-145, miR-149, miR-182, miR-194, miR-758) was assessed by quantitative RT-PCR analysis, and concentration of chosen proteins (Fn and FPN1) was estimated by ELISA. In this study we identified a set of discriminating genes in tumor and normal colon tissues and evaluated their association with clinicopathological characteristics of CRC patients. Now we are going to investigate whether this difference is also observed in serum.

## Materials and methods

### Collection of tissue samples

Paired samples of primary CRC and adjacent normal mucosa (5–10 cm away from the tumor) were collected during surgery from 73 patients in accordance with protocols approved by the committee on the Ethics of Research in Human Experimentation at the Medical University of Lodz (Poland), and informed consent was obtained in accordance with the Declaration of Helsinki. Among patients were 38 men and 35 women. Their mean age was 63.1 years (SD 10.4 years, range 44–81 years). Based on the grading system, the numbers of patients with well (G1), moderately (G2), and poorly differentiated (G3) cancer cells were 21, 39, and 11, respectively, and in 2 cases the differentiation could not be evaluated. According to pTNM staging system of the American Joint Committee on Cancer (AJCC), the numbers of patients in stage I, IIA, IIIA, IIIB, IIIC and IV were 26, 12, 9, 11, 9, and 4, respectively. In 2 cases the assessment of invaded lymph nodes was not feasible. The patient’s gender, age, tumor location, local invasion, differentiation and hemoglobin levels were obtained from surgical and pathological records. All samples were stored at −80 °C.

### RNA extraction

Isolation of total RNA from frozen tumor and non-tumor tissues was performed with *mir*Vana™ miRNA Isolation Kit (Ambion) according to manufacturer’s recommendations. RNA was eluted with 30 μL of nuclease free water. The concentration of RNA was detected with Picodrop. RNA quality was determined using 2100 Bioanalyzer (Agilent Technologies).

### Reverse transcriptase reactions

The stem-loop RT primers for miRNA were purchased from Applied Biosystems. TaqMan^®^ MicroRNA Reverse Transcription Kit (Applied Biosystems) was used to synthesize cDNA of miRNA, according to the guidelines provided by the manufacturer. 10 ng of total RNA was added to the reaction tube to make up a final volume of 15 μL reaction mix and incubated (30 min, 16 °C and 30 min, 42 °C) in Thermocycler (Biometra). The reverse transcriptase was inactivated (5 min, 85 °C). To obtain cDNA from mRNA, 200 ng of the extracted RNA was reversely transcribed in a final volume of 20 μL with AccuScript *PfuUltra* II RT-PCR Kit (Stratagene), according to manufacturer’s instructions. Samples were incubated (10 min, 25 °C and 30 min, 42 °C) and held at −20 °C.

### Real time PCR (QPCR): detection of mRNA level with SYBR Green

Real-time PCR was performed on Stratagene Mx3005P instrument (Stratagene). The primer sequences of target genes and the reference gene ribosomal protein L13a (RPL13A) are given in Table 1. PCR reaction was carried out using Brilliant II SYBR^®^ Green QPCR Master Mix (Stratagene). 10 ng of cDNA and 100 nM primers were added to the reaction tube to make up a final volume of 25 μL reaction mix. The following run protocol was used: denaturation program (95 °C, 10 min) and amplification and quantification programs repeated 40 times (95 °C, 15 s and 57 °C, 1 min). Melting curves were generated for each real-time RT-PCR to verify the specificity of PCR reaction. All samples were amplified simultaneously in triplicate in a single run. Relative quantification of mRNA was determined by comparative Ct method [29]. The mRNA level of tested gene was presented as the amount normalized to RPL13A value, and was calculated as $2^{{ - \Updelta {\text{C}}_{\text{t}} }}$, while the relative expression analysis of target gene was presented as the n-fold change in gene expression normalized to the reference gene and relative to the control, and was calculated as $2^{{ - \Updelta \Updelta {\text{C}}_{\text{t}} }}$.

**Table 1:** Primer sequences used in QPCR and PCR reactions

Gene	Forward 5′–3′	Reverse 5′–3′	Product size (bp)
DMT1	TCCACCATGACAGGAACCTA	TGGCAATAGAGCGAGTCA	105
Fn	GGAGAGGGAACATGCTGAGA	GCACACTCCATTGCATTCAG	126
FPN1	ACAGCAGTCTACGGGCTGGT	CTGTACCACCAGCGAGGTCT	117
HAMP	CACAACAGACGGGACAACTT	CGCAGCAGAAAATGCAGATG	129
Heph	TTAAGCCCTCTCACCGTCATCA	GCCAGAACAACGAGCAGAAGG	170
HFE	GATCATGAGAGTCGCCGTGT	ATGTGATCCCACCCTTTCAG	107
HJV	CTGCCTACATTGGCACAACT	CCTTGATGGAGAAGGAGAGC	66
IRP1	TGCTTCCTCAGGTGATTGGCTACA	TAGCTCGGTCAGCAATGGACAACT	171
IRP2	ACCAGAGGTGGTTGGATGTGAGTT	ACTCCTACTTGCCTGAGGTGCTTT	102
Tf	AGCAGAGACCACCGAAGACT	AGACAAACCCTCCATCCAAG	80
TfR1	GGTTGCAAATGCTGAAAGC	AAGGAAGGGAATCCAGGTGT	147
TfR2	GTGGACCGACACGCACTAC	TAGACGTCAGGGTCCTCCAG	123
RPL13A	CCTGGAGGAGAAGAGGAAAGAGA	TTGAGGACCTCTGTGTATTTGTCAA	126
HAMP-exon1	CGGTCCCAGACACCAGAGCAAGC	CCCTGCTGCCCTGCTAAGGACC	192
HAMP-exons2-3	GCCATCCTCTGCACCCCCTTCTG	GGAAGGGAGGGGACGGGGGCA	320

### Real time PCR (QPCR): detection of miRNA level with TaqMan probes

Real-time PCR was performed on Stratagene Mx3005P instrument (Stratagene). Brilliant QPCR Master Mix (Stratagene) and 1 ng of cDNA were used in each PCR reaction. Primers for miRNA genes were purchased from Applied Biosystems. The U6 small nuclear RNA served as an endogenous control for miRNA amplification. The reaction was performed at 95 °C for 10 min, followed by 40 amplification cycles at 95 °C for 15 s and 60 °C for 1 min. All samples were amplified simultaneously in triplicate in a single run. Relative quantification of miRNA was determined by comparative Ct method.

### Tissue homogenates preparation

Tumor and nontumor tissues were rinsed in ice-cold PBS (0.02 mol/L, pH 7.2) to remove excess blood thoroughly. 25 mg of tissue was homogenized with a TissueRuptor (QIAGEN) in 1 mL of PBS (0.01 mol/L, pH 7.4) supplemented with protease inhibitor cocktail tablets (Roche). The resulting suspension was subjected to two freeze–thaw cycles to further break the cell membranes. After that, the homogenates were centrifugated (5 min, 5,000×*g*) and the total protein in supernates was quantified with Micro BCA™ Protein Assay Kit, according to manufacturer’s recommendations (ThermoSCIENTIFIC). The supernates were stored at −20 °C.

### ELISA

The concentration of Fn and FPN1 in tumor and nontumor specimens was estimated with Human Fn (FE) ELISA Kit (BMASSAY) and Enzyme-linked Immunosorbent Assay Kit For FPN1 (USCNK Life Science Inc.), respectively, according to manufacturer’s recommendations. Human Actin Beta (ACTb) ELISA Kit (BMASSAY) was used as an amount control. Samples were measured photometrically at 450 nm with a microplate reader (Multiskan Ascent, Thermo Labsystems). The relative amount of protein was normalized to the control, and calculated as tumor/nontumor concentration ratio.

### DNA extraction

Tumor and nontumor tissues were homogenized with TissueRuptor (QIAGEN) in lysis buffer. After incubation (15 min, 55 °C) 10 μL of RNase was added to the samples and incubated (1 h, 55 °C). Subsequently the samples were incubated with 10 μL of proteinase K (1 h, 55 °C) and then extraction was performed. 0.5 mL of phenol:chloroform (1:1) mix was added and samples were centrifuged (3 min, 13,000×*g*). Supernatant was transferred to fresh tubes and 0.5 mL of chloroform:isoamyl alcohol (24:1) mix was added. After centrifugation (3 min, 13,000×*g*) supernatant was transferred to fresh tubes and 100 μL of 10 M ammonium acetate and 1 mL of ethanol 96 % were added. Samples were frozen (−80 °C) and centrifuged (10 min, 13,000×*g*). Pellets were washed with cold ethanol 96 %, samples were centrifuged (10 min, 13,000×*g*) and pellets were resuspended with 50 μL of LoTE buffer (3 mM Tris–HCl (pH 7.5), 0.2 mM EDTA) and frozen (−80 °C).

### DNA sequencing analysis

Amplification of three exons of *HAMP* gene was conducted using primers listed in Table 1. Starter binding to complementary DNA matrix sites was carried out at 58 °C. The PCR product sequences were established by automatic DNA sequencing. The occurrence of single nucleotide polymorphisms was analyzed with Chromas and CLUSTALW Software.

### Statistical analysis

All calculations were performed with the STATISTICA StatSoft Version 10. Data were ln-transformed to obtain a normal distribution. Paired Student’s *t* test was used to evaluate the difference between paired samples (tumor and normal tissues) and one-way ANOVA followed by Tukey’s test was used to compare the differences among groups. Correlation between the expression of miRNAs, mRNAs and protein concentration was confirmed using the Pearson’s correlation coefficient analyses. Discriminant analysis was used to find a combination of genes which characterizes different clinicopathological features in colorectal adenocarcinoma patients. Statistical significance was assumed to be *p* < 0.05.

## Results

### Expression of iron-related genes in CRC and normal mucosa specimens

Previous studies reported, that some of iron-related genes are overexpressed in colorectal adenocarcinoma [27, 28]. To investigate the alterations in expression of genes involved in iron metabolism or its regulation, we examined over 20 genes. Among them 12 encoded proteins (*DMT1*, *Fn*, *FPN1*, *HAMP*, *Heph*, *HFE*, *HJV*, *IRP1*, *IRP2*, *Tf*, *TfR1*, *TfR2*) and nine encoded miRNAs (miR-19a, miR-31, miR-133a, miR-141, miR-145, miR-149, miR-182, miR-194, miR-758). Sequences of these miRNA genes are homological to 3′-UTRs of several iron-related genes (Table 2), according to the DIANA Database, and thus analyzed miRNAs are considered to play a role in regulation of iron metabolism.

**Table 2:** Prediction of miRNA targets

miRNA	miRNA sequence 3′–5′	Target genes	Position of target 3′UTR	Seed match
miR-19a	AGUCAAAACGUAUCUAAACGUGU	DMT1	89–95	7mer-1A
miR-31	UCGAUACGGUCGUAGAACGGA	TfR1	527–533	8mer
miR133a	GUCGACCAACUUCCCCUGGUUU	Fn (light chain)	114–120	8mer
miR-141	GGUCGAAAUGGUCUGUCACAAU	TfR1	1,462–1,468	7mer-m8
miR-145	UCCCUAAGGACCCUUUUGACCUG	TfR1	1,027–1,033	7mer-1A
miR-149	CCCUCACUUCUCUGCCUCGGUCU	DMT1	35–41	7mer-m8
miR-182	UCACACUCAAGAUGGUAACGGUUU	TfR1	810–816	7mer-1A
miR-194	AGGUGUACCUCAACGACAAUGU	TfR1	1,439–1,435	7mer-1A
	AGGUGUACCUCAACGACAAUGU	FPN1	21–27	7mer-m8
miR-758	CCAAUCACCUGGUCCAGUGUUU	TfR1	1,323–1,329	7mer-m8

Underlined letters are the homological sequence of miRNAs to target genes. The homology of both RNAs concerns only 7-nucleotide sequence and not the whole sequence of miRNA as is observed in siRNAs

QPCR assay revealed, that all analyzed mRNAs were detectable in both tumor and normal tissues tested, and *Fn* mRNA was highly expressed in adenocarcinoma as well as in control specimens, but not significantly different in these tissues (Table 3). Most of analyzed mRNAs, except *Heph*, *IRP1* and *Tf*, were expressed more frequently in tumor than in normal tissues, but difference between both specimens reached significance (*p* < 0.05) only in 3 of 12 analyzed mRNAs (*FPN1*, *IRP1* and *Tf*). Among this three genes *FPN1* was up-regulated in CRC (*p* = 0.039), and *IRP1* and *Tf* were down-regulated in comparison to adjacent normal tissue (*p* = 0.046 and *p* = 0.017, respectively).

**Table 3:** mRNA or miRNA level determined in tumor and normal tissues by QPCR reactions

Gene	Control (normal tissue)		Sample (tumor tissue)		Statistical significance
	Mean	SD	Mean	SD	
DMT1	−7.131	0.81	−6.979	1.10	*p* = 0.389
Fn	0.079	1.01	0.211	0.94	*p* = 0.600
FPN1	−6.203	2.60	−5.482	2.09	*p* = 0.039
HAMP	−8.082	1.30	−7.548	1.77	*p* = 0.082
Heph	−4.195	2.16	−4.538	2.34	*p* = 0.158
HFE	−6.254	1.97	−6.173	2.28	*p* = 0.640
HJV	−7.562	2.90	−6.812	2.44	*p* = 0.166
IRP1	−7.028	2.15	−7.350	2.13	*p* = 0.046
IRP2	−7.340	1.39	−7.315	1.68	*p* = 0.979
Tf	−7.632	1.49	−8.535	1.59	*p* = 0.017
TfR1	−7.516	1.18	−7.291	1.50	*p* = 0.397
TfR2	−8.719	2.77	−8.426	2.91	*p* = 0.182
miR-19a	−2.590	1.09	−2.353	1.07	*p* = 0.140
miR-31	−6.435	1.05	−4.078	1.37	*p* < 0.001
miR-133a	0.098	1.54	−2.106	2.14	*p* < 0.001
miR-141	−2.000	1.66	−0.697	1.45	*p* < 0.001
miR-145	3.226	1.44	1.398	2.36	*p* < 0.001
miR-149	−4.732	1.48	−6.092	1.32	*p* < 0.001
miR-182	−7.410	1.67	−6.123	1.73	*p* < 0.001
miR-194	−1.623	1.65	−0.747	2.38	*p* = 0.041
miR-758	−7.152	0.58	−7.225	0.69	*p* = 0.876

Contrary to mRNAs, almost all examined miRNA genes were expressed at higher levels in tested materials. The difference between miRNA levels in both tumor and normal tissues were highly significant (*p* < 0.001) in most genes encoding miRNAs, except miR-19a and miR-758, in which the level of mRNA remained almost the same (Table 3). The expression intensities of miR-31, miR-141, miR-182 and miR-194 in tumor tissues were significantly enhanced, and miR-133a, miR-145 and miR-149 were decreased in comparison to those in adjacent normal tissues, according to RT-QPCR data.

### Alterations in genes expression in various clinicopathological stages of CRC patients

Different expression of analyzed genes, especially encoding miRNAs, was observed in patients suffering from colorectal adenocarcinoma. To estimate, whether these alterations appear either in early stage of disease or depend on the progression of CRC, we compared the expression of analyzed genes in various clinicopathological features of this disease, including histological grade, depth of invasion, nodal status, tumor location, diagnosis, and also gender and age of patients.

**Fig. 1:**
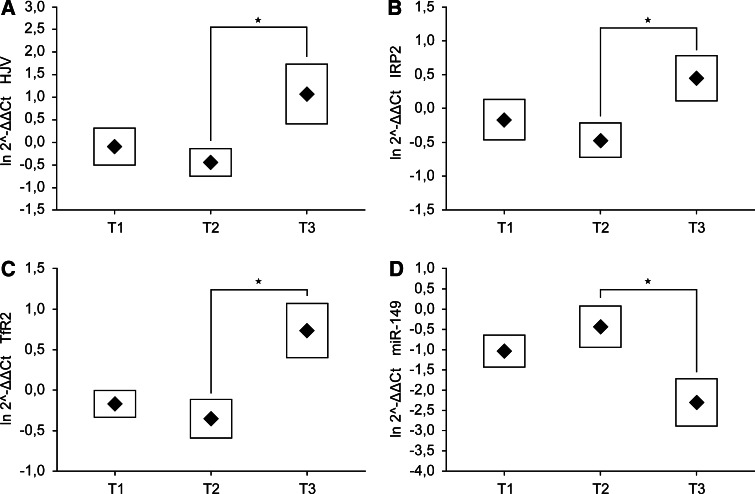
Expression of **a** *HJV*, **b** *IRP2*, **c** *TfR2* and **d** miR-149 genes in T1, T2 and T3 stages of colorectal adenocarcinoma. Data presented as mean ± SE for the relative expression of analyzed genes calculated as ${ \ln }\; 2^{{ - \Updelta \Updelta {\text{C}}_{\text{t}} }}$ . Significance of differences between T2 and T3 stages, estimated with one-way ANOVA and post hoc Tukey’s test for *HJV*, *IRP2*, *TfR2* and miR-149 genes was *p* = 0.049*, p* = 0.041*, p* = 0.016 and *p* = 0.028, respectively

**Fig. 2:**
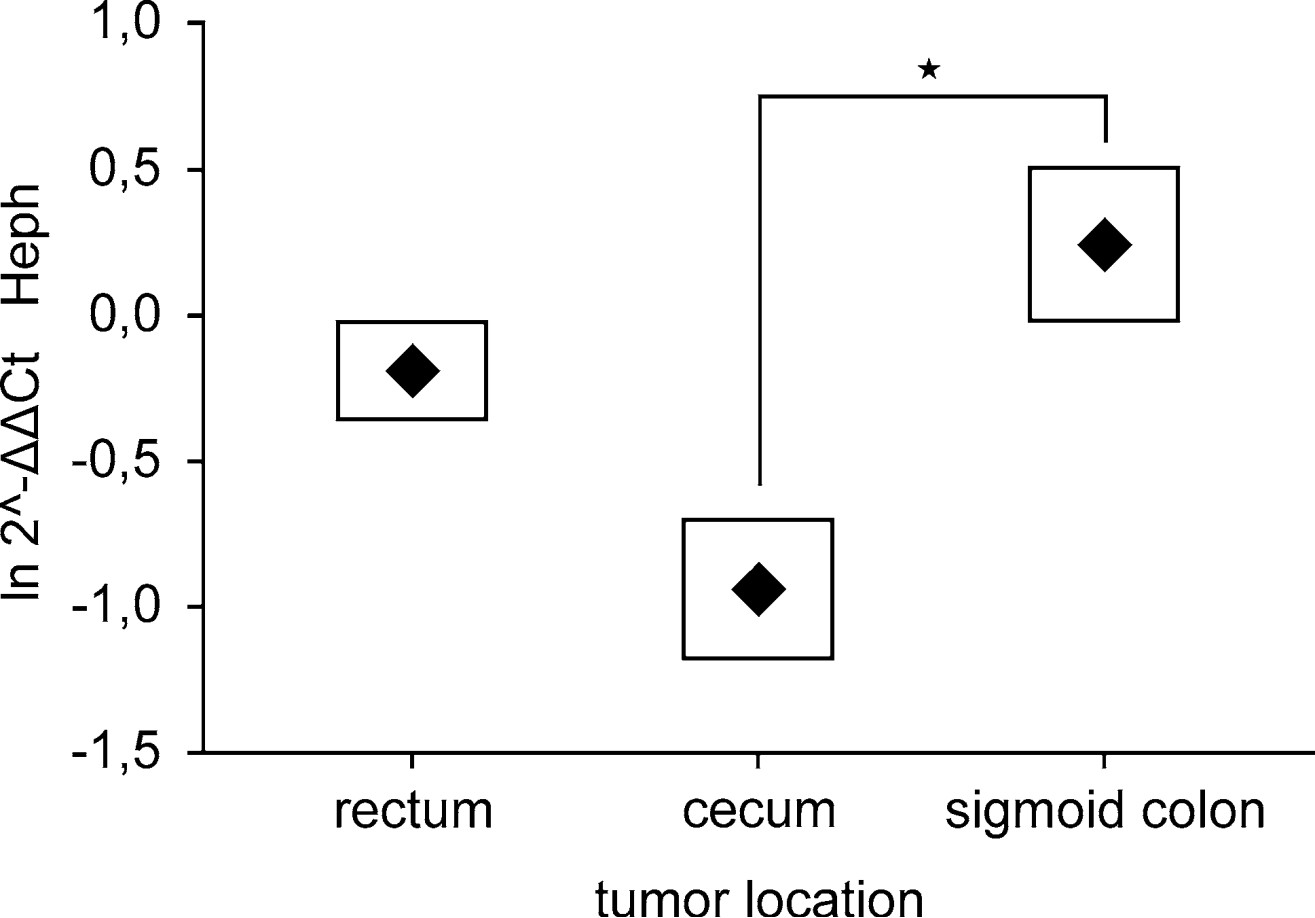
Expression of *Heph* gene in tumors localized in various parts of colon. Data presented as mean ± SE for the relative expression of analyzed gene calculated as ${ \ln }\; 2^{{ - \Updelta \Updelta {\text{C}}_{\text{t}} }}$. Significance of differences, estimated with one-way ANOVA and post hoc Tukey’s test was *p* = 0.040

According to QPCR data, the expression profiles of *HJV*, *IRP2*, *TfR2* and miR-149 genes were dependent on depth of tumor invasion. *HJV*, *IRP2* and *TfR2* were up-regulated and mir-149 gene was down-regulated in T3 stage of CRC in comparison to T1 and T2 stages (Fig. 1). There was no statistically significant difference in the expression of remained iron-related genes among various T stages. Additionally, the *Heph* demonstrated higher expression in sigmoid colon than in cecum or rectum as was shown in Fig. 2, whereas the tumor location remained without significant influence for the expression of other tested genes. Moreover, we compared the expression of iron-related genes in male and female populations and found, that the *Tf* was significantly (*p* = *0.009*) overexpressed in women. The data did not reveal any additional alterations in mRNA or miRNA levels in dependence on cancer cell differentiation, nodal invasion, diagnosis or patients age.

### Alteration in ferroportin concentration in colorectal adenocarcinoma

**Fig. 3:**
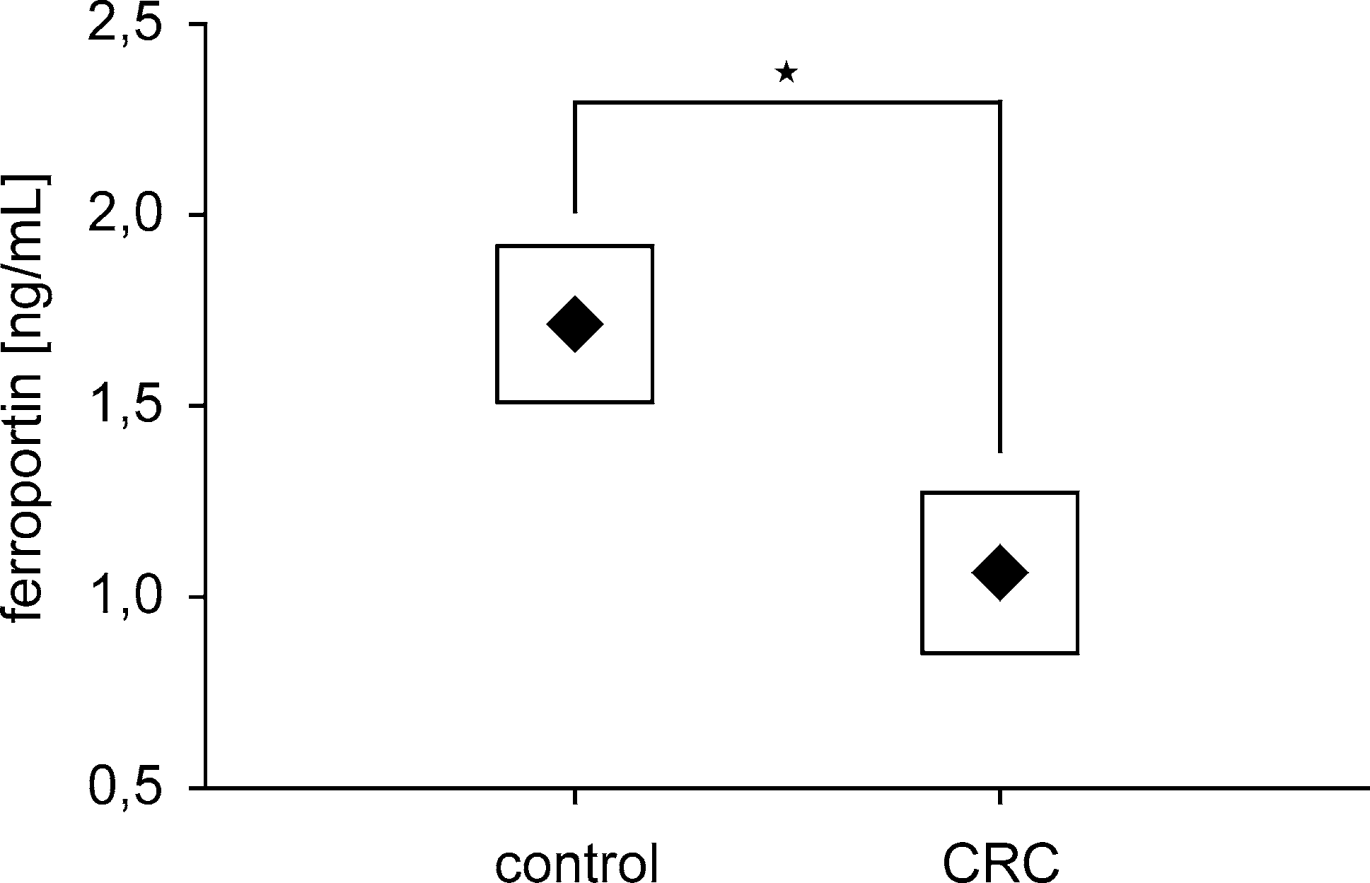
Concentration of ferroportin in tumor and normal tissue, assessed by ELISA assay. Data presented as mean ± SE, and significance of differences, estimated with Wilcoxon signed-rank test was *p* = 0.023, *n* = 73

To analyze, whether the alterations in gene expression were observed at the protein level, we estimated the concentration of two proteins Fn and FPN1, which can be regulated by IRPs and/or miRNAs levels. Both proteins were detectable in tumor as well as in normal tissues tested. The high concentration of Fn, reaching 300 ng/mL in some cases, was observed in adenocarcinoma and adjacent normal tissues as well, but the difference in Fn level between these paired specimens was not significant. Contrary to this, the amount of FPN1 was significantly higher (*p* = *0.023*) in normal than in tumor tissues (Fig. 3).

### Genes involved in CRC progression

**Fig. 4:**
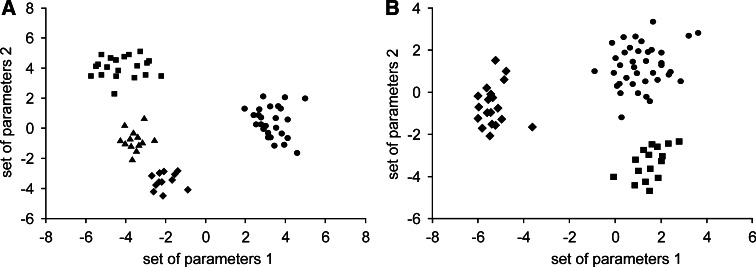
Discriminant analysis of patients **a** in various stages of CRC, according to pTNM system or **b** with different diagnosis of cancer. **a** In this model 7 variables were used (*DMT1*, *HAMP*, *TfR2*, miR-31, miR-149, miR-182, miR-194); Wilks’ lambda was 0.006, *p* < 0.0001, *n* = 71; Stage I (*circles*), Stage IIA (*diamonds*), Stages IIIA + IIIB (*squares*), Stages IIIC + IV (*triangles*). **b** In this model 9 variables were used (*DMT1*, *IRP1*, *IRP2*, *Tf*, miR-31, miR-133a, miR-145, miR-149, miR-194); Wilks’ lambda was 0.046, *p* < 0.0006, *n* = 73; Adenocarcinoma tubulare (*circles*), Adenocarcinoma tubulare/mucinosum (*squares*), Adenocarcinoma mucinosum (*diamonds*)

To identify genes, which can play a role in progression of colorectal adenocarcinoma or discrimination of CRC patients in dependence on clinicopathological characteristics, we conducted discriminant analysis of iron-related gene expression. The forward stepwise mode constructed discriminant functions including all genes under study, and showed that *DMT1*, *HAMP*, *TfR2*, miR-31, miR-149, miR-182, miR-194 were the most significant parameters responsible for progression of colorectal adenocarcinoma, assigning all cases correctly (*p* < *0.0001*). Fig. 4a shows that the alterations in expression of chosen genes can distinguish patients with the wide spectrum of cancer stages.

Moreover, the stepwise forward discriminant functions including nine variables can correctly discriminate (*p* < 0.0006) all colorectal adenocarcinoma cases. Forward stepwise mode showed that *DMT1*, *IRP1*, *IRP2*, *Tf*, miR-31, miR-133a, miR-145, miR-149, miR-194 were the most significant parameters responsible for proper diagnosis. This set of genes was differently expressed in adenocarcinoma tubulare, adenocarcinoma tubulare/mucinosum and adenocarcinoma mucinosum (Fig. 4b).

### Correlation between mRNAs expression levels in colorectal adenocarcinoma

It was shown, that IRPs can control the posttranscriptional expression of genes by stabilization and protection of *TfR1* mRNA from endonucleolytic degradation or inhibition of the H- and L-*Fn* mRNAs translation into protein in liver and macrophages [20]. To investigate whether this regulation is also observed in CRC, we estimated the levels of *IRP1* and *IRP2* mRNA and correlated them with *TfR1* and *Fn* mRNA levels, and also with expression levels of two other genes (*DMT1* and *FPN1*) containing IREs in UTRs. Correlation coefficients values of mRNA levels, which reached the statistical significance at various stages and different histopathological grades of CRC were shown in Tables 4 and 5.

**Table 4:** Correlation between relative expression of mRNAs or mRNA vs. miRNA in patients with well and moderately differentiated cancer cells

mRNA vs. mRNA or mRNA vs. miRNA	G1		G2	
	Correlation coefficient	Statistical significance	Correlation coefficient	Statistical significance
IRP1 vs. DMT1	r = 0.73	*p* = 0.039	r = 0.24	*p* = 0.330
IRP1 vs. TfR1	r = 0.73	*p* = 0.038	r = 0.29	*p* = 0.024
IRP2 vs. DMT1	r = 0.67	*p* = 0.067	r = 0.53	*p* = 0.021
IRP2 vs. TfR1	r = 0.84	*p* = 0.009	r = 0.72	*p* < 0.001
HAMP vs. FPN1	r = −0.08	*p* = 0.848	r = 0.51	*p* = 0.035
HAMP vs. HFE	r = −0.22	p = 0.605	r = 0.52	*p* = 0.029
HAMP vs. HJV	r = −0.20	*p* = 0.641	r = 0.56	*p* = 0.015
HFE vs. HJV	r = 0.71	*p* = 0.050	r = 0.77	*p* < 0.001
TfR1 vs. miR-145	r = −0.06	*p* = 0.884	r = −0.58	*p* = 0.011

**Table 5:** Correlation between relative expression of mRNAs or mRNA vs. miRNA in patients with different stages of colorectal adenocarcinoma

mRNA vs. mRNA or mRNA vs. miRNA	Stage I		Stage IIA		Stages IIIA + IIIB		Stages IIIC + IV	
	Correlation coefficient	Statistical significance	Correlation coefficient	Statistical significance	Correlation coefficient	Statistical significance	Correlation coefficient	Statistical significance
IRP1 vs. DMT1	r = 0.67	*p* = 0.012	r = 0.74	*p* = 0.157	r = −0.17	*p* = 0.752	r = −0.31	*p* = 0.687
IRP1 vs. FPN1	r = 0.38	*p* = 0.221	r = 0.05	*p* = 0.939	r = −0.77	*p* = 0.002	r = −0.52	*p* = 0.023
IRP2 vs. DMT1	r = 0.36	*p* = 0.233	r = 0.87	*p* = 0.056	r = 0.71	*p* = 0.012	r = 0.63	*p* = 0.026
IRP2 vs. TfR1	r = 0.84	*p* < 0.001	r = 0.75	*p* = 0.147	r = 0.66	*p* = 0.153	r = 0.48	*p* = 0.515
IRP2 vs. TfR2	r = 0.67	*p* = 0.012	r = 0.20	*p* = 0.743	r = 0.87	*p* = 0.023	r = 0.76	*p* = 0.043
HAMP vs. HFE	r = 0.44	*p* = 0.136	r = −0.07	*p* = 0.916	r = 0.79	*p* = 0.018	r = 0.79	*p* = 0.042
HAMP vs. HJV	r = 0.59	*p* = 0.032	r = 0.15	*p* = 0.813	r = 0.55	*p* = 0.259	r = 0.69	*p* = 0.306
HFE vs. HJV	r = 0.60	*p* = 0.030	r = 0.74	*p* = 0.013	r = 0.86	*p* = 0.028	r = 0.71	*p* = 0.085
HFE vs. Tf	r = 0.66	*p* = 0.015	r = 0.07	*p* = 0.913	r = 0.40	*p* = 0.433	r = −0.62	*p* = 0.380
HFE vs. TfR1	r = 0.68	*p* = 0.011	r = 0.74	*p* = 0.151	r = 0.75	*p* = 0.087	r = 0.55	*p* = 0.453
HFE vs. TfR2	r = 0.31	p = 0.306	r = 0.34	p = 0.577	r = 0.82	p = 0.044	r = 0.81	p = 0.189
FPN1 vs.miR-194	r = 0.29	*p* = 0.369	r = 0.34	*p* = 0.571	r = −0.89	*p* < 0.001	r = −0.51	*p* = 0.034
TfR1 vs. miR-31	r = −0.33	*p* = 0.265	r = 0.56	*p* = 0.325	r = 0.86	*p* = 0.029	r = 0.51	*p* = 0.048
TfR1 vs. miR-145	r = −0.37	*p* = 0.219	r = −0.09	*p* = 0.884	r = −0.87	*p* = 0.025	r = −0.64	*p* = 0.041

Strong positive correlation was observed between *TfR1* and both *IRP* mRNA levels in well and moderately differentiated cancer cells. *IRP2* expression profile was also significantly associated with relative expression of both genes encoding *TfR* in patients with stage I of cancer and additionally with *TfR2* transcript level in more advanced stages of this disease. Moreover, *IRP1* expression level was also positively correlated with *DMT1* mRNA level in early stage of disease as well as in first grade of cancer, however in patients having lymph nodes invaded by the tumor *IRP1* was negatively associated with relative expression of gene encoding other transporter protein FPN1. In contrast, *IRP2* mRNA level was positively correlated with *DMT1* mRNA level in more invasive stages of CRC and in moderately differentiated tumor cells.

Iron regulatory mechanism is also modulated by hepcidin level, which expression is related to expression of *HFE* or *HJV* genes in liver [14, 15]. To investigate, whether this mechanism is also observed locally in CRC we correlated *HAMP* mRNA level with *HFE* and *HJV* mRNA expression levels and also with *FPN1*, which is a receptor for hepcidin. Association between *HAMP* and *FPN1* mRNA levels was found in moderately differentiated cancer cells. Also positive correlation between *HAMP* and *HFE* mRNA levels was observed in more advanced stages and grades of this disease. Contrary, association between relative expression of genes encoding hepcidin or its second regulatory protein hemojuvelin was shown to be significant in early stage of CRC, as well as in grade 2 of cancer. Moreover, association between both hepcidin regulatory genes (*HFE* and *HJV*) was found in all grades and stages of this disease, except stages IIIB and IV of cancer. Additionally, *HFE* mRNA level was positively correlated with *Tf* as well as with *TfR1* mRNA in stage I of CRC and also with *TfR2* in more advanced stages of this disease.

### Correlation between mRNA expression and homologous miRNA levels in CRC

Other regulatory mechanism of iron metabolism is depended on presence or absence of miRNAs in the tissue. All analyzed miRNAs, according to DIANA Database, are homological to 3′-UTRs of several iron-related genes (Table 2). We analyzed the correlation between miRNAs and the expression of target genes levels in various grades of CRC and in different stages of this disease, and the correlation coefficients values, which reached the statistical significance were listed in Tables 4 and 5.

*TfR1* expression level was positively correlated with miR-31 and inversely associated with miR-145 levels in more advanced stages of colorectal adenocarcinoma presenting higher nodal status. *TfR1* expression level was also negatively correlated with miR-145 level in moderately differentiated but not with well differentiated cancer cells. Strong inverse correlation was also observed between levels of *FPN1* mRNA and miR-194 in patients having lymph nodes invaded by the tumor. Remained miRNAs, which were analyzed in this study were not associated with their target genes in any stage and grade of CRC.

### Correlation between mRNA or miRNA expression and protein level in tumor tissue

**Fig. 5:**
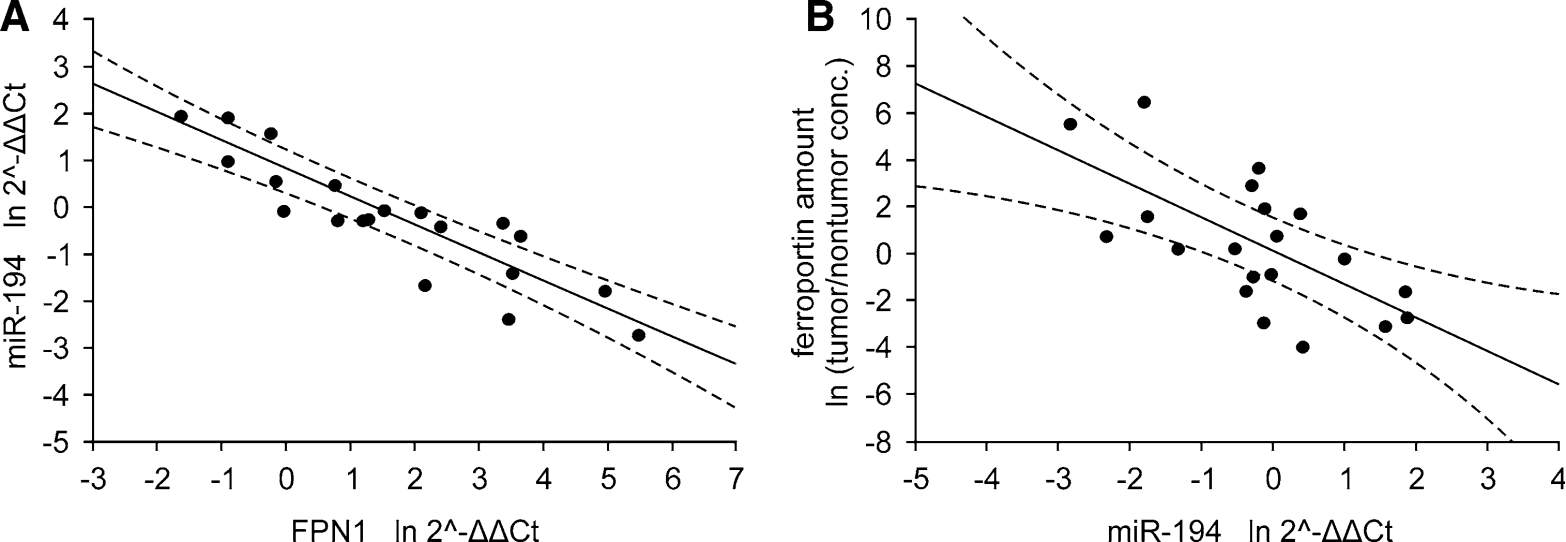
Correlation between miR-194 level and **a** FPN1 mRNA or **b** ferroportin levels in patients with IIIA and B stages of CRC. Data presented as relative expression of analyzed genes calculated as ${ \ln }\; 2^{{ - \Updelta \Updelta {\text{C}}_{\text{t}} }}$ or relative amount of protein calculated as ln tumor/nontumor concentration ratio. Correlation coefficients and statistical significance were estimated with Pearson’s correlation coefficient analyses and were r = −0.89*, p* < 0.001 for *FPN1* mRNA vs. miR-194, r = *−*0.66*, p* = 0.002 for miR-194 versus ferroportin, *n* = 20. *Dashed lines* determine 95 % confidence interval

**Fig. 6:**
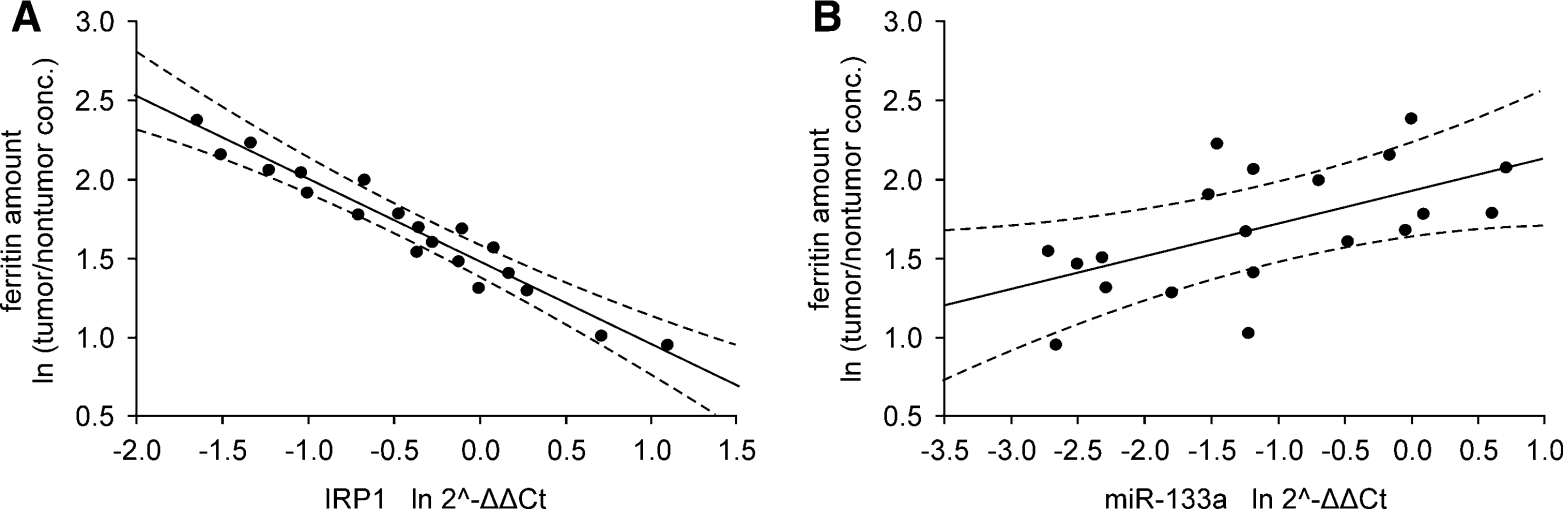
Correlation between ferritin level and **a** IRP1 mRNA or **b** miR-133a levels in patients with IIIA and B stages of CRC. Data presented as relative expression of analyzed genes calculated as ${ \ln }\; 2^{{ - \Updelta \Updelta {\text{C}}_{\text{t}} }}$ or relative amount of protein calculated as ln tumor/nontumor concentration ratio. Correlation coefficients and statistical significance were estimated with Pearson’s correlation coefficient analyses and were r = −0.97*, p* < 0.001 for IRP1 mRNA vs. ferritin, r = 0.56*, p* = 0.011 for miR-133a vs. ferritin, *n* = 20. *Dashed lines* determine 95 % confidence interval

To further investigate, whether those correlations can be confirmed at the protein level, we estimated the concentration of ferritin and ferroportin in tumor and normal specimens. Strong negative correlation (r = −0.66*, p* = 0.002) in IIIA and IIIB stages of CRC was observed between FPN1 and miR-194 levels (Fig. 5), but not with FPN1 and *IRP1* mRNA levels. However, concentration of this transporter was not associated with its regulatory molecules in early stages of this disease. Similarly to FPN1, the level of other analyzed protein Fn was negatively associated with *IRP1* mRNA level but positively correlated with miR-133a level (r = −0.97*, p* < 0.001 and r = 0.56*, p* = 0.011, respectively) in patients having lymph nodes invaded by the tumor, which was shown in Fig. 6.

### *HAMP* gene mutations in tumor tissues

We performed sequence analyzes of HAMP gene in all tumor and normal specimens and identified mutations in the heterozygous state in 4 patients. Two individuals carried *HAMP* R59G missense mutation, which was previously described [30]. The effect of this mutation is to prevent the formation of the 25–amino acid mature peptide [31], which causes severe hereditary hemochromatosis. Two other mutations (S4R and Q6E) were novel and localized in the first exon of *HAMP* gene encoding C-terminal peptide, which is cut down during the maturation process of hormone, and probably these alterations do not influence the maturation process of hepcidin hormone. Because of small amount of cases with *HAMP* mutations, the evaluation of mutation effect on gene expression was not possible.

## Discussion

Proper regulation of iron metabolism is critical for many important metabolic processes including DNA synthesis, oxidative cell metabolism, haemoglobin synthesis and cell respiration. Alternations of iron level disturb the body homeostasis by forming reactive oxygen species (ROS) responsible for oxygen stress generation, that leads to many clinical disorders including carcinogenic events [32, 33]. A body of evidence suggest, that high dietary iron intake is a major risk factor for CRC [2, 3]. Diet is thought to be the aspect of particular interest in the pathogenesis of CRC since it has been proven to have direct influence on composition of intestinal lumen, and through that on the colonic mucosa [34]. Moreover, it has been shown that body iron stores were positively associated with the development of precancerous lesions in the colon, colonic adenoma or polyps [35]. Posttranscriptional regulation of iron-related genes in liver, enterocytes and macrophages has been well characterized, and is associated with the presence of either IREs [20–22], or sequences homological for miRNAs in UTRs [23] of several genes involved in iron metabolism. However, the local role of these genes in tumor tissues was poorly investigated. We hypothesized, that there can be alterations in expressing profile of some genes involved in iron metabolism promoting the accumulation of iron in colonocytes, and this could be an additional factor contributing to malignant transformation and tumor progression.

We have shown, that all analyzed iron-related genes encoding proteins are detectable in both tumor and normal tissues tested. *Fn* mRNA and its protein product are highly expressed in all stages of adenocarcinoma, as well as in control specimens, which can suggest that normal colonocytes and neoplastic colon cells can accumulate high levels of iron. We investigated the correlations between Fn and expression levels of several genes (*IRP1*, *IRP2* and miR-133a), which can play a role in the posttranscriptional regulation of gene encoding Fn in CRCs. We have shown, that *IRP1* mRNA level is inversely associated with Fn amount only in more invasive stages of CRC, while miR-133a is positively correlated with Fn concentration in the same individuals. It can suggest, that IRP1 could be involved in regulation mechanism of Fn amount, however the role of miR-133a in this process remains unclear, since miRNAs are considered rather as suppressors than activators of translation process. Perhaps, there is some other mechanism responsible for regulation of gene encoding Fn, which could play a role not only in more advanced stages of this disease.

Since colorectal tissues can collect elevated amount of iron, we have assessed the mRNA level of iron transporters in 73 patients with CRC. Our findings confirmed previous observations, that genes encoding DMT1 and TfR1 are more often up-regulated in tumor than in normal tissues [27], and we attempted to identify the mechanism responsible for this regulation. Since IRPs can be involved in posttranscriptional regulation of *DMT1* [21], we assessed the correlation between *DMT1* and *IRP*s mRNAs levels. Our experiments revealed, that in early stage of CRC the level of *DMT1* transcript is well associated with *IRP1* mRNA level, and in more advanced stages of disease with *IRP2* transcript level. It suggests, that *DMT1 *mRNA could be stabilized by both IRPs during the successive progression of neoplastic process. Simultaneously, IRP2 could be engaged in the mRNA stabilization of other iron transporter TfR1 in early stage of disease, as it was shown with the correlation analyzes of mRNA levels. However, in more advanced stages of CRC, mRNA level of *TfR1* is related to miR-31 level, which suggests, that during the cancer progression *TfR1* gene could be silenced due to binding of its transcript to homological sequences of miR-31. We have shown that *IRP*s mRNA levels are correlated in various stages of colorectal adenocarcinoma with relative expression of genes encoding all transporters involved in iron metabolism. It can suggest that some interactions among proteins involved in iron metabolism observed in liver and macrophages can also take place in tumor tissue of this type of cancer.

In contrast to earlier data, our experiments indicated, that *FPN1* mRNA is significantly elevated in CRC, but the level of its product ferroportin was significantly reduced. It was shown that *FPN1* expression is regulated at several levels, including posttranscriptional regulation by the IRE in 5′-UTR bound to IRPs [22], and posttranslational regulation by the action of hepcidin, in response to increased iron abundance [11]. miR-194 was also considered to play a role in regulation of *FPN1* expression by targeting the 3′-UTR of the transcript, leading to suppression of translation or rapid degradation of target mRNA, and thus, regulation of iron metabolism. For the first time we have shown, that *FPN1* mRNA level is significantly and negatively associated with the level of miR-194, causing the reduction of transporter amount in tumor tissues of patients with more advanced stages of CRC. Indeed, assessment of FPN1 concentration in examined tissues confirmed this finding, which provided novel information on the mechanism of microRNA-induced iron metabolism regulation. This observation is also supported by the fact, that in the same individuals there is a strong inverse correlation between *FPN1* and *IRP1* mRNAs levels, which can imply that during the cancer progression the translation of mRNA into protein can be less sufficient due to increased amount of inhibitors of this process. However, the protein level was not correlated with *IRP1* mRNA level, which can suggest that this inhibitory mechanism is not so effective as that previously described. Simultaneously, we have not observed the correlation of *FPN1* mRNA level with the amount of colorectal hepcidin mRNA in patients with more invasive CRC stages, although expression of *HAMP* gene tended to be up-regulated in tumor tissues, as was previously described in small population of patients [28]. It may suggest, that this tendency of *HAMP* overexpression is related with up-regulation of *HFE* and *HJV* genes in tumors of the same individuals, which is confirmed with correlation analysis of both transcripts levels. Thus, reduction of ferroportin amount in tumor tissue, as well as overexpression of genes responsible for iron intake can entail the accumulation of iron in neoplastic cells, leading to increase of Wnt signaling, which has been shown to be crucial in colorectal carcinogenesis [36].

Since miRNAs play a significant role in many fundamental cellular processes such as cell differentiation, growth, proliferation, and apoptosis, their expression levels differ between healthy and cancerous tissues. Indeed, expression profile studies of miRNAs exhibited their possible tumor suppressor or oncogenic function. To enrich this findings, we assessed the expression levels of over 300 human miRNAs in 5 % of examined CRC patients. For further investigation we have chosen genes, which are homological to 3′-UTRs of iron-related genes, according to the DIANA Database, and reveal altered expression profile. Among them, miR-31, miR-141, miR-182 and miR-194 are up-regulated, and miR-133a, miR-145 and miR-149 are down-regulated. Some of our results are in agreement with previous observations, where it was shown that miR-133a and miR-145 were consistently reduced and miR-31 and miR-182 were regularly overexpressed in colorectal neoplasm in comparison to healthy colon mucosa [37–41], but these alterations are not associated with the stage of CRC. However, we have also shown, that miR-19a expression level is not significantly different in adenocarcinoma in comparison to control specimens, which is in opposite to previously described data showing overexpression of this gene in CRC patients [38]. Interestingly, the alterations in expressing profile of miR-141, miR-149 and mir-194 in adenocarcinoma specimens have never been documented yet and are the new findings on this field. In addition, the expression level of miR-149 is correlated with the depth of tumor invasion. These alterations are observed even in the early stage of disease, which can suggest, that miR-31, miR-133a, miR-141, miR-145, miR-149, miR-182 and miR-194 could be a potential set of markers for early detection of colorectal adenocarcinoma, but the level of this mRNAs in serum has not been investigated yet.

Until recently, the pathologist has been limited in regard to the classification and prognostication of CRCs to morphologic and pathologic staging of the tumor. Several statistical analyses from our study supported the conclusion that expression of genes involved in regulation or mechanism of iron metabolism can differentiate patients with various stages of colorectal adenocarcinoma. We identified a set of genes (*DMT1*, *HAMP*, *TfR2*, miR-31, miR-149, miR-182, miR-194), which expression intensities are altered during the progression of disease. These parameters can explain all analyzed cases assigning them correctly in dependence of CRC stage. Our findings suggest, that at the level of colorectal tumor tissue iron metabolism could be, at least partially, locally regulated, leading to accumulation of iron and thus promoting cancer progression. If so, these genes could become potential prognostic indicators for patients with CRC.
